# Usability and Perceived Usefulness of the AFib 2gether Mobile App in a Clinical Setting: Single-Arm Intervention Study

**DOI:** 10.2196/27016

**Published:** 2021-11-19

**Authors:** Alok Kapoor, Anna Hayes, Jay Patel, Harshal Patel, Andreza Andrade, Kathleen Mazor, Carl Possidente, Kimberly Nolen, Rozelle Hegeman-Dingle, David McManus

**Affiliations:** 1 Department of Medicine University of Massachusetts Chan Medical School Worcester, MA United States; 2 Division of Medical Affairs Pfizer Inc New York, NY United States

**Keywords:** shared decision-making, mobile health, stroke risk, anticoagulation risk, anticoagulation, atrial fibrillation, anticoagulation therapy, atrial flutter, mobile phone

## Abstract

**Background:**

Although the American Heart Association and other professional societies have recommended shared decision-making as a way for patients with atrial fibrillation (AF) or atrial flutter to make informed decisions about using anticoagulation (AC), the best method for facilitating shared decision-making remains uncertain.

**Objective:**

The aim of this study is to assess the AFib 2gether mobile app for usability, perceived usefulness, and the extent and nature of shared decision-making that occurred for clinical encounters between patients with AF and their cardiology providers in which the app was used.

**Methods:**

We identified patients visiting a cardiology provider between October 2019 and May 2020. We measured usability from patients and providers using the Mobile App Rating Scale. From the 8 items of the Mobile App Rating Scale, we reported the average score (out of 5) for domains of functionality, esthetics, and overall quality. We administered a 3-item questionnaire to patients relating to their perceived usefulness of the app and a separate 3-item questionnaire to providers to measure their perceived usefulness of the app. We performed a chart review to track the occurrence of AC within 6 months of the index visit. We also audio recorded a subset of the encounters to identify evidence of shared decision-making.

**Results:**

We facilitated shared decision-making visits for 37 patients visiting 13 providers. In terms of usability, patients’ average ratings of functionality, esthetics, and overall quality were 4.51 (SD 0.61), 4.26 (SD 0.51), and 4.24 (SD 0.89), respectively. In terms of usefulness, 41% (15/37) of patients agreed that the app improved their knowledge regarding AC, and 62% (23/37) agreed that the app helped clarify to their provider their preferences regarding AC. Among providers, 79% (27/34) agreed that the app helped clarify their patients’ preferences, 82% (28/34) agreed that the app saved them time, and 59% (20/34) agreed that the app helped their patients make decisions about AC. In addition, 32% (12/37) of patients started AC after their shared decision-making visits. We audio recorded 25 encounters. Of these, 84% (21/25) included the mention of AC for AF, 44% (11/25) included the discussion of multiple options for AC, 72% (18/25) included a provider recommendation for AC, and 48% (12/25) included the evidence of patient involvement in the discussion.

**Conclusions:**

Patients and providers rated the app with high usability and perceived usefulness. Moreover, one-third of the patients began AC, and approximately 50% (12/25) of the encounters showed evidence of patient involvement in decision-making. In the future, we plan to study the effect of the app on a larger sample and with a controlled study design.

**Trial Registration:**

ClinicalTrials.gov NCT04118270; https://clinicaltrials.gov/ct2/show/NCT04118270

**International Registered Report Identifier (IRRID):**

RR2-21986

## Introduction

### Significance

Atrial fibrillation (AF) and atrial flutter occur in epidemic proportions in the United States, affecting approximately 5 million people [1-5]. As AF is a major risk factor for stroke, professional societies recommend anticoagulation (AC) in most patients; however, some patients are reluctant to start taking it. Limited health literacy, inaccurate perception of the risk of AF, and lack of trust in physicians contribute to patient refusal, with up to 39.3% (257,415/655,000) of patients remaining off AC across the country [6-8]. Among the patients who start AC, many discontinue treatment, especially after the onset of bleeding or other setbacks. Providers struggle to evaluate the risks and benefits of AC [8]. The American Heart Association and other professional societies recommend shared decision-making as a way of arriving at the optimal decision about AC for each patient; however, the feasibility of integrating shared decision-making into routine clinical care is unclear [9-11].

### Approach

The AFib 2gether mobile app [12] was developed by Pfizer Inc in consultation with a cardiologist (DM) as a potential approach for operationalizing shared decision-making around AC for AF. Specifically, Pfizer Inc convened a hackathon over a 2-day period that included physicians, pharmacists, app developers, and legal and patient education professionals at its headquarters. During this meeting, analysts programmed and improved the app using an iterative design methodology. Using the app, we aim to promote collaboration between patients and providers. Specifically, by using the app, patients first identify their stroke risk factors and later receive a stroke risk score with the projected yearly stroke risk. Patients may then select (from a list of commonly asked examples developed during the hackathon) the questions that they would like their provider to answer at their next visit. The provider can review patient entries on the same app (but with different landing pages based on their role as provider) at the time of each visit to help the patient make an informed decision about AC. The app has not been previously tested with patients and providers for usability, perceived usefulness, frequency of AC starts occurring after visits in which providers and patients use the app, or evidence of shared decision-making. We recently published a protocol to evaluate the usability, perceived usefulness, and feasibility of the app for actual clinical encounters between patients with AF and their cardiology providers [13]. This paper reports the results from the completed study and their interpretation.

## Methods

### Overview

We previously published a protocol for integrating the AFib 2gether app in encounters between patients and their cardiology providers [13]. We briefly summarize the methods below. The sponsor of this work, Pfizer Inc, reviewed and edited the drafts of our study design and manuscript interpreting the results.

### Population

The study included patients with AF and with a CHA_2_DS_2_-VASc score ≥2 who were not currently on AC and were visiting cardiology providers at the University of Massachusetts Memorial Medical Center. The CHA_2_DS_2_-VASc score assigns 1 point for congestive heart failure, hypertension, age 65-74 years, diabetes, vascular disease history such as myocardial infarction, and female sex and 2 points for age ≥75 years or prior stroke or transient ischemic attack.

### Procedures

We obtained consent from the cardiology providers. After enrollment, each cardiology provider completed questionnaires regarding their knowledge and confidence in AF management. Specifically, we asked providers to report their confidence in using information about stroke risk and bleeding risk to determine appropriate antithrombotic therapy and familiarity with the American College of Cardiology/American Heart Association/Heart Rhythm Society (ACC/AHA/HRS) guidelines for AF management. We also asked 2 questions requiring providers to accurately calculate the CHA_2_DS_2_-VASc score and make a decision to prescribe AC based on it for a patient presented in a clinical vignette. Next, we moved to recruit the patients of these providers by mailing introductory letters with a fact sheet to facilitate verbal consent. We then telephoned the patients to obtain their consent and enroll them. During the enrollment telephone call, we asked patients to download the app if they had a smartphone and provided a brief orientation (lasting 2-3 minutes, although we did not record the exact duration). For those without a smartphone, we provided a study smartphone when the patient arrived for their in-person visit with their cardiology provider.

After the app orientation, patients answered questions in the app to determine their stroke risk score based on the CHA_2_DS_2_-VASc risk score. Once the participants answered the questions, the app displayed their stroke risk factors and allowed them to select questions from a list of commonly asked questions that they wanted to ask their providers during their visits based on this score. The app then sent the patients' completed inventory of stroke risk factors and questions for discussion to their providers to review. The participants then completed the Mobile App Rating Scale (MARS) questionnaire [14]. The providers then reviewed their patients’ stroke risk answers and compared them to what they knew about the patients’ risk. After the providers made any corrections to the stroke risk factors, the patient scores were made available to the patients and providers to review and discuss.

At the visit, we reminded the providers to review the app information completed by the patients (if they had not done so before the start of the visit). We then audio recorded the conversation for those who had in-person visits (ie, those who were seen before restrictions were put in place during the COVID-19 pandemic of 2019-2020). At the conclusion of the visit, both the patients and providers completed a 3-question survey on the perceived usefulness of the app.

We transcribed the audio recordings and reviewed them for evidence of shared decision-making using a prespecified list of items modified from an established instrument for measuring shared decision-making (ie, Observing Patient Involvement in Decision Making Scale) [15] as well as novel items specifically related to AF management (Table 1).

**Table 1:** Providers’ perceived usefulness of the AFib 2gether app (N=34).

Usefulness item			Frequency (encounters),^a^ n (%)
**The app improved my understanding of the patient’s preferences regarding AC^b^**			
	Strongly agree or agree	27 (79)	
	Neutral	7 (21)	
	Disagree or strongly disagree	0 (0)	
**The app will save me time in focusing on those items which are most important to patients**			
	Strongly agree or agree	28 (82)	
	Neutral	5 (15)	
	Disagree or strongly disagree	1 (3)	
**The app will help me decide if my patient needs to be on AC**			
	Strongly agree or agree	20 (59)	
	Neutral	12 (35)	
	Disagree or strongly disagree	2 (6)	

^a^Providers contributed multiple times to the frequency statistics, as they answered our survey after each shared decision-making visit. In 3 cases, we were not able to collect responses from providers.

^b^AC: anticoagulation.

### Descriptive Statistics

We collected demographic information and information on comorbid conditions from electronic capture of patient information from our institution’s electronic health record (EHR). Specifically, we worked with an experienced EHR analyst who interrogated the Clarity database associated with Epic Systems EHR to identify demographic information. Through the use of ICD codes that we previously validated, the analyst was also able to identify the presence of comorbid conditions [3,16]. From chart reviews, we also captured the reason why a patient was not on AC. In this review, we grouped patients into the following categories: low AF burden, refused AC, fall risk, gastrointestinal bleeding, other bleedings, or unspecified reasons. During our review, we also captured the number of years elapsed since AF onset. For providers, we administered a provider knowledge survey, as described in the protocol.

### Outcomes

#### Primary Outcomes

We grouped items in the MARS into the 3 domains of functionality, esthetics, and overall quality or the number of stars out of 5, following a validated protocol [17]. The perceived usefulness questions for patients spanned 3 usefulness domains: improving knowledge, clarifying preferences, and making a decision about AC. Similarly, the provider usefulness questions spanned 3 items: clarifying patient preferences, saving time, and helping to make a decision for the patient. The response format for each set of questions was on a 5-point Likert scale, ranging from strongly agree to strongly disagree. Given the small numbers, we consolidated *strongly agree* with *agree* and *strongly disagree* with *disagree*.

#### Secondary Outcomes

We tracked the start of (although not the adherence to) AC in the 6 months after the shared decision-making visit. From the audio recordings, we counted the number of turns of conversations dedicated to discussions about AC. Each turn represented a dialog from 1 speaker before turning to the other. In terms of the evidence of shared decision-making, we captured how often a provider presented medication options and discussed the benefits and risks of AC and how often patients participated in the discussions, following an established instrument from which we adapted our review process. [18]. We also collected some app-specific information on whether the provider checked the risk score on the app and whether the patients’ self-identified risk factors were correct.

### Analysis

For each domain of the usability of the app or the MARS items, we calculated the mean and SD. By contrast, for perceived usefulness, we grouped patients into 3 categories, combining *strongly agree* with *agree* and *strongly disagree* with *disagree*. For the start of AC, we tabulated the frequency and calculated the simple percentage of the number of starts divided by the total number of patients.

We received approval for our study from the University of Massachusetts Chan Medical School Institutional Review Board. We conducted our study ethically by obtaining informed consent, with no change in treatment for patients who declined to participate.

## Results

### Descriptive Statistics

We sent letters to or approached in person a total of 165 eligible patients. From this pool of 165 patients, we conducted visits using our app with 37 (22.4%) patients who were seeing 13 cardiology providers. Of the 128 patients who were not included, we were unable to reach 10 (7.8%). Another 23.4% (30/128) declined, with not being interested as the most common reason, followed by not feeling well enough to participate as the next most common reason. For the remaining 68.8% (88/128) of patients, no attempt was made, as the study staff were not available or did not need to recruit additional patients at the time of the patient’s visit. Nearly all patients (36/37, 97%) were White, and approximately half of the enrolled participants were aged ≥75 years. This age distribution followed the typical epidemiology of AF, including those in our previous studies [3,16,19]. Most patients were men (26/37, 70%) and had a CHA_2_DS_2_-VASc score between 2 and 4 (28/37, 76%). A chart review of the reasons why patients were not on AC before their appointment revealed that, of the 37 patients, 16 (43%) patients had a low AF burden, whereas 10 (27%) patients refused without further explanation clearly documented. In terms of the length of AF diagnosis, 41% (15/37) of patients had a history of AF in the past 1 to 5 years (Table 2). All patients entered enough information in the app to compute their CHA_2_DS_2_-VASc scores. Of the 37 patients, 21 (57%) asked at least one question. Of the 37 patients, 21 (57%) did not have their own smartphone and therefore required the use of our study phone.

**Table 2:** Comparison of key patient characteristics (N=37).

Characteristics			Frequency, n (%)
**Age** **(years)**			
	>75	17 (46)	
	65-74	14 (38)	
	<65	6 (16)	
**Sex**			
	Female	11 (30)	
	Male	26 (70)	
**Race**			
	Non-White	1 (3)	
	White	36 (97)	
**Ethnicity**			
	Hispanic	1 (3)	
	Non-Hispanic	36 (97)	
**Individual predictors of CHA_2_DS_2_-VASc score^a^**			
	Congestive heart failure	14 (38)	
	Hypertension	33 (89)	
	Diabetes	7 (19)	
	Stroke or transient ischemic attack	5 (14)	
	Vascular disease	14 (38)	
**CHA_2_DS_2_-VASc score**			
	2	9 (24)	
	3	10 (27)	
	4	9 (24)	
	5	6 (16)	
	6	2 (5)	
	7	0 (0)	
	8	0 (0)	
	9	1 (3)	
**Reason for not being on anticoagulation before index appointment with cardiology provider^b^**			
	Low AF^c^ burden	16 (43)	
	Refused	10 (27)	
	Not listed	5 (14)	
	Fall risk	1 (3)	
	Gastrointestinal bleeding	2 (5)	
	Other bleeding	3 (8)	
**Years since AF onset**			
	1-5	15 (41)	
	5-10	12 (32)	
	>10	10 (27)	
**Had another AF episode in the past year**			
	Yes	26 (70)	

^a^The CHA_2_DS_2_−VASc score assigns 1 point for congestive heart failure, hypertension, age 65-74 years, diabetes mellitus, vascular disease history, such as myocardial infarction, and female sex and 2 points for age >75 years, previous stroke, or transient ischemic attack.

^b^Index appointment is the encounter in which we used the AFib 2gether app.

^c^AF: atrial fibrillation.

Most enrolled providers had been in practice for ≥10 years. Of the 13 providers, 9 (69%) were physicians, 2 (15%) were physician assistants, and 2 (15%) were nurse practitioners. The providers’ self-rated confidence was high in our sample. More specifically, 85% (11/13) of providers were very confident in assessing antithrombotic therapy for patients who had stroke and selecting the appropriate AC, and 77% (10/13) felt very confident in using oral AC therapies for reducing stroke risk. The HAS-BLED bleeding risk score assigns 1 point for hypertension, abnormal renal function, abnormal liver function, tendency for bleeding, labile international normalized ratio, age >65 years, history of alcohol or drug usage, and medication usage predisposing to bleeding. Confidence in using the HAS-BLED bleeding risk calculator was more variable. Only 31% (4/13) of providers felt very confident in this skill. For a 2-part knowledge inquiry based on a clinical vignette presented, 77% (10/13) of providers accurately calculated a CHA_2_DS_2_-VASc score and based anticoagulant decision-making on it. Of the 13 providers, 7 (54%) providers reported that <25% of their patients had a diagnosis of AF, whereas 6 (46%) reported that >25% had a diagnosis of AF (Table 3).

**Table 3:** Provider characteristics, knowledge, and confidence in managing patients with AF^a^ (N=13).

Demographics		Frequency, n (%)
**Years in practice**		
	<10	3 (23)
	10-20	4 (31)
	>20	6 (46)
**Type of provider**		
	Nurse practitioner	2 (15)
	Physician assistant	2 (15)
	MD^b^	9 (70)
**How confident are you in assessing antithrombotic therapy for stroke risk patients?**		
	Somewhat confident	0 (0)
	Moderately confident	2 (15)
	Very confident	11 (85)
**How confident are you in selecting appropriate anticoagulant therapy?**		
	Somewhat confident	0 (0)
	Moderately confident	2 (15)
	Very confident	11 (85)
**How confident are you in using oral** **-** **anticoagulant therapies for reducing stroke risk?**		
	Somewhat confident	0 (0)
	Moderately confident	3 (23)
	Very confident	10 (77)
**How confident or familiar are you in using CHA_2_DS_2_-VASc scores^c^ to assess stroke risk?^d^**		
	Somewhat confident	0 (0)
	Moderately confident	2 (17)
	Very confident	10 (83)
**How confident are you in applying ACC/AHA/HRS^e^ guidelines to the management of AF?^f^**		
	Somewhat confident	0 (0)
	Moderately confident	3 (23)
	Very confident	10 (77)
**How confident or familiar are you in using HAS-BLED^g^ score to assess bleeding risk?**		
	Somewhat confident	2 (15)
	Moderately confident	7 (54)
	Very confident	4 (31)
**Correctly identified CHA_2_DS_2_-VASc score=3 for a clinical vignette of a 73-year-old male patient with hypertension and CHF^h^**		
	Correct	10 (77)
	Incorrect	3 (23)
**Correctly identifying that aspirin would not be an appropriate antithrombotic for the above patient**		
	Correct	13 (100)
	Incorrect	0 (0)
**Approximately what percentage of your adult patients have a diagnosis of AF?**		
	<25%	7 (54)
	>25%	6 (46)

^a^AF: atrial fibrillation.

^b^MD: doctor of medicine.

^c^The CHA_2_DS_2_‐VASc score assigns 1 point for congestive heart failure, hypertension, age 65-74 years, diabetes mellitus, vascular disease history such as myocardial infarction, and female sex and 2 points for age >75 or previous stroke or transient ischemic attack.

^d^For this item, N=12, given nonresponse from 1 provider.

^e^ACC/AHA/HRS: American College of Cardiology/American Heart Association/Heart Rhythm Society.

^f^Refers to the 2014 jointly issued guidelines from the American College of Cardiology, American Heart Association, and Heart Rhythm Society that provide guidance on the use of anticoagulation for patients with AF.

^g^HAS-BLED score is a bleeding risk score that includes predictors for hypertension, abnormal renal or liver function, stroke, bleeding history or predisposition, labile international normalized ratio, older adults, and drugs or alcohol concomitantly.

^h^CHF: congestive heart failure.

### Primary Outcomes

Patients rated the AFib 2gether app highly on all domains of usability. The MARS combined average functionality score for patients was 4.51 (SD 0.61) out of a possible score of 5. Patients also rated the app highly in the MARS esthetics category with a score of 4.26 (SD 0.51) out of 5. Patients rated the app usability highly; the mean MARS star usability rating was 4.24 (SD 0.89) out of a possible 5. There was no sizable difference in the rating of patients who asked questions and those who did not (overall rating 4.30 vs 4.17).

Patients also gave high perceived usefulness ratings. Specifically, 40% (15/37) of patients agreed with the statement “the app improved my knowledge regarding anticoagulation.” Similarly, 62% (23/37) of patients agreed with the statement “the app helped me clarify my provider preferences regarding anticoagulation” (Table 4).

**Table 4:** Patients’ perceived usefulness of the app (N=37).

Usefulness item		Frequency, n (%)
**The app improved my knowledge regarding AC^a^**		
	Strongly agree or agree	15 (40)
	Neutral	18 (49)
	Disagree or strongly disagree	4 (11)
**The app clarified my AC preferences to my provider**		
	Strongly agree or agree	23 (62)
	Neutral	11 (30)
	Disagree or strongly disagree	3 (8)
**The app helped me decide if I should go on AC**		
	Strongly agree or agree	20 (54)
	Neutral	12 (32)
	Disagree or strongly disagree	5 (14)

^a^AC: anticoagulation.

Provider ratings for usability were also high. Out of a maximum of 5, the mean of providers’ ratings for functionality was 4.19 (SD 0.50), for esthetics was 4.04 (SD 0.50), and for overall star-based quality was 3.76 (SD 0.44).

Providers also reported high perceived usefulness. Specifically, 79% (27/34) somewhat or strongly agreed that the app helped clarify the preferences of their patients, and 82% (28/34) agreed that the app saved them time. Slightly fewer providers (20/34, 59%) agreed that the app helped their patients make a decision about AC (Table 1).

### Secondary Outcomes

Approximately 32% (12/37) of patients started AC after their appointment. This included 56% (9/16) of patients who were previously not on AC because of the low AF burden. Of the 10 patients who had previously refused to be on AC, 2 (20%) started AC after the visit. Of the 5 patients without a specified reason for not being on AC before the visit, 1 (20%) started AC after it.

We were able to collect audio recordings from the first 68% (25/37) of patients we recruited. After that point, our institution restricted in-person recruitment to limit the spread of COVID-19 in 2019. From the available encounters, we noted that AC for AF was mentioned 84% (21/25) of the time. We also noted the discussion of multiple options of AC in 44% (11/25) of patient encounters. In 72% (18/25) of the encounters, the provider made a recommendation regarding AC for the patient. The recommendations included whether the provider believed that the patient should be anticoagulated, as well as which anticoagulant the provider believed would be best for the patient. We identified that in 48% (12/25) of the patient encounters, there was evidence of patient involvement in the discussion (Table 5).

**Table 5:** Frequency of shared decision-making or AF^a^ management items observed in audio recordings of patient encounters (N=25).

General theme and specific item or shared decision-making element		Frequency, n (%)
**Background**		
	AF mentioned	24 (96)
	Mention of AC^b^ for AF in the conversation	21 (84)
**Medication options**		
	Multiple options for AC mentioned	11 (44)
	Provider makes a recommendation regarding AC	18 (72)
**Stroke and bleeding risk and risk factors**		
	CHA_2_DS_2_-VASc stroke risk score^c^ mentioned by physician	6 (24)
	Evidence that the provider shared the stroke risk with the patient	14 (56)
	Bleeding risk addressed by provider (patient can bring up the issue so long as the provider tries to give an answer)	12 (48)
	Bleeding risk used for the purpose of deciding whether to prescribe AC	7 (28)
	Bleeding risk factors addressed in terms of identifying factors that are modifiable—alcohol, previous labile INR,^d^ hypertension, and aspirin or NSAID^e^ use	11 (44)
**AC benefits**		
	Discussion included benefits of AC	2 (8)
**AC resumption**		
	Discussion of AC resumption after bleeding	13 (52)
**Patient involvement**		
	Evidence of patient involvement in the discussion (eg, patient declined AC and patient wanted to discuss with [another person])	12 (48)
	Patient asked a question or multiple questions	10 (40)
	Provider checked that the patient understood all the information they told them (eg, information about AC and AF status)	4 (16)
	Provider offered patient explicit opportunities to ask questions during the decision-making process	5 (20)
**App-specific items**		
	Provider checked the risk score on the app	12 (48)
	Patient’s self-identified risk factors were correct according to provider and it was mentioned during the encounter	12 (48)
	Patient selected questions in the app	11 (44)

^a^AF: atrial fibrillation.

^b^AC: anticoagulation.

^c^The CHA_2_DS_2_−VASc score assigns 1 point for congestive heart failure, hypertension, age 65-74 years, diabetes mellitus, vascular disease history such as myocardial infarction, and female sex and 2 points for age >75 years or previous stroke or transient ischemic attack.

^d^INR: international normalized ratio.

^e^NSAID: nonsteroidal anti-inflammatory drug.

## Discussion

### Principal Findings

Most patients in this study gave high usability ratings to our shared decision-making app across 3 separate domains. They also reported that the app helped clarify their preferences to their providers and improved their knowledge of AC. One-third of these patients started AC after their appointment with their providers. Approximately half of the patients demonstrated involvement in AC decision-making.

To understand the importance of our findings, we compared them with findings of other intervention studies in the AC management of AF. Man-Son-Hing et al [20] developed a decision aid based on a risk stratification scheme that helped patients with AF and their providers make informed decisions about whether to use warfarin or aspirin. They then tested this in a randomized trial. They found that patients in the group assigned to the decision aid were more able to make definite choices regarding antithrombotic therapy (99% vs 94%; *P*=.02). More recently, Kunneman et al [21] tested a shared decision-making tool that provided individualized risk estimates of stroke in various anticoagulant treatment options. Although they did not find a significant effect on treatment decisions, more clinicians were satisfied with the encounter in the intervention arm compared with the standard arm. Neither of the 2 studies specifically studied the usability or perceived usefulness of their shared decision-making tool.

Our app compared favorably with the ratings published for other health mobile technology tools evaluated using MARS. In particular, authors of a systematic review documented an average score (averaged across the same 3 domains of usability that we analyzed) ranging from 2.40 to 2.63 for blood pressure apps deployed on smartphone platforms [22]. We found that our app performed significantly better than those included in the review of blood pressure apps, although our particular cohort of patients did not evaluate other apps’ limiting comparisons.

Our findings have several implications. Our app appears to be usable by both patients and providers. Moreover, 32% (12/37) of patients started AC after having used the app for their clinical visit. To better understand the effectiveness of the app, we will require a controlled study, given that the participants who agreed to participate in our study may have been more educated or *activated* than typical patients in routine clinical practice. In terms of other implications, we also found that there was a moderately high level of patient involvement as measured through audio recordings. However, some elements of shared decision-making occurred infrequently. Further enhancements of the app, for example, prompts to encourage the use of more shared decision-making elements, may stimulate even greater encouragement for patient involvement. Other enhancements to the app may also better prepare patients to participate in AC discussions. Of the 37 patients, 16 (43%) were not on AC because of the isolated and low burden of AF. However, the current ACC/AHA/HRS guidelines for AC do not take the AF burden into account when recommending treatment [10]. Further clarification of guidelines by its authors for prescribing AC in the setting of isolated and low burden of AF and education of providers may help overcome some of the gaps in AC use beyond patient hesitancy or refusal.

### Limitations

There are a number of limitations to this study. One limitation is the absence of a control group. Nevertheless, we are not aware of other single-arm studies that demonstrated a 32% increase in AC with a single-encounter intervention. Expanding our testing to other centers and including a control group would provide better information on the potential benefits of the app. Another limitation of our work is that we were only able to record encounters for 68% (25/37) of patients, given the interruption in our study caused by the restrictions against in-person recruitment at the time of the COVID-19 pandemic. Despite this limitation, we demonstrated a moderately high level of patient involvement in the visits that we recorded. Another limitation was the absence of custom information for patients with discrete reasons, such as bleeding, for not being on AC at the time of the visit. Future iterations of the app may want to include custom information. Finally, our population was heterogeneous in terms of the reason for not being on AC, potentially introducing bias into the measurements we recorded.

### Conclusions

In conclusion, patients and providers found the AFib 2gether app usable, and there was a high level of perceived usefulness that facilitated an informed discussion with the provider, leading to increased guideline-based AC management. We await further testing at other centers and in a controlled study design to assess the potential benefit of the app and its ability to increase AC use, consistent with prevailing professional society guidelines designed to prevent stroke in patients with AF.

## Abbreviations

AC: anticoagulation
ACC/AHA/HRS: American College of Cardiology/American Heart Association/Heart Rhythm Society
AF: atrial fibrillation
EHR: electronic health record
MARS: Mobile App Rating Scale
